# Successful pregnancy in maple syrup urine disease: a case report and review of the literature

**DOI:** 10.1186/s12937-018-0357-7

**Published:** 2018-05-12

**Authors:** Sarah Catharina Grünert, Stefanie Rosenbaum-Fabian, Anke Schumann, Karl Otfried Schwab, Nadja Mingirulli, Ute Spiekerkoetter

**Affiliations:** Department of General Pediatrics, Adolescent Medicine and Neonatology, Medical Center – University of Freiburg, Faculty of Medicine, Mathildenstraße 1, 79106 Freiburg, Germany

**Keywords:** Maple syrup urine disease, MSUD, Pregnancy, Leucine tolerance, Protein tolerance, Branched-chain amino acids, Obstetrics, Metabolic disease

## Abstract

**Background:**

Maple syrup urine disease (MSUD) is an autosomal recessive disorder of branched-chain amino acid metabolism. Patients with MSUD are at risk of life-threatening metabolic decompensations with ketoacidosis and encephalopathy. These episodes are often triggered by physiological stress. Only few cases of pregnancies in MSUD mothers have been reported so far.

**Case presentation:**

We present the favorable outcome of a pregnancy in a woman with classical MSUD. She presented in the metabolic outpatient clinic in week 7 of gestation. Branched-chain amino acid concentrations were measured at least weekly to adjust dietary leucine intake. Despite excellent compliance, leucine concentrations frequently exceeded the target value of < 300 μmol/L during the first trimester. From the second trimester until delivery, protein and leucine intake increased continuously to about threefold compared to pre-pregnancy values. To maximize patient safety during delivery and the postpartum period, a detailed plan including peripartal infusion therapy, dietary recommendations and monitoring parameters was developed. Primary Caesarean section was performed in week 38 of gestation, and the patient gave birth to a healthy girl. Lactation was successfully implemented. Leucine levels were maintained within the target range throughout the complete postpartum period.

In addition to our case, we give an overview about all cases of pregnancies in MSUD mothers published so far.

**Conclusions:**

Management of pregnancy, delivery, postpartum period and lactation may be challenging in patients with MSUD. Careful monitoring and interdisciplinary collaboration is essential to minimize the risk of metabolic crisis, especially after delivery.

**Electronic supplementary material:**

The online version of this article (10.1186/s12937-018-0357-7) contains supplementary material, which is available to authorized users.

## Background

Maple syrup urine disease (MSUD, OMIM 248600) is an autosomal recessive inborn error of branched-chain amino acid (BCAA) metabolism caused by deficiency of the branched-chain α-ketoacid dehydrogenase complex. Mutations in 3 of four genes that encode the catalytic subunits of the enzyme complex (E1, E2, E3) have been described. MSUD is a rare disease in most populations, with incidence estimates of 1:185,000 live births [1, 2]. Much higher incidences are found in certain Mennonite populations due to a founder variant resulting in a frequency for classic MSUD as high as approximately one in 380 live births [3]. Diagnosis is based on the detection of markedly elevated concentrations of BCAA and allo-isoleucine in plasma and urine as well as branched-chain ketoacid analogues in urine of affected individuals. Patients with MSUD are prone to metabolic crises, presenting with ketoacidosis, vomiting, poor feeding, neurological symptoms and encephalopathy, often triggered by catabolism due to intercurrent infections, surgery or excessive intake of BCAA. The toxic metabolite is leucine, and high concentrations are associated with ketosis and the risk of cerebral edema [4]. Although most patients manifest within the neonatal period, metabolic crises can occur at any age. Long-term treatment of MSUD includes a low-protein diet with supplementation of a BCAA-free amino acid mixture.

Only few cases of pregnancies in MSUD patients have been reported so far [4–10]. The overall outcome seems to be favorable. Nevertheless, pregnancy can put patients at risk for decompensation due to possible hyperemesis gravidarum, catabolic stress during delivery and excessive protein turnover during the postpartum period. We report the successful outcome of a pregnancy in a 26-year-old woman with classical MSUD and our experiences in the management from pregnancy to lactation. Additionally, we provide a review of the literature including all cases of pregnancies in MSUD patients published so far.

## Case presentation

The patient is a 26-year-old woman who was diagnosed with classical MSUD presymptomatically within the first days of life due to an older affected brother. She has been on a low-protein diet with supplementation of a BCAA-free amino acid mixture since birth with good metabolic control and normal intellectual development. A number of short hospitalizations had been necessary during childhood because of mild metabolic decompensations during intercurrent illnesses. The highest leucine concentration ever measured was 1145 μmol/L within the neonatal period. Leucine tolerance prior to pregnancy was 700-800 mg per day. Genetic analysis revealed a homozygous mutation in the *BCKBHD* gene, c.721A > T, p.K241*.

**Fig. 1:**
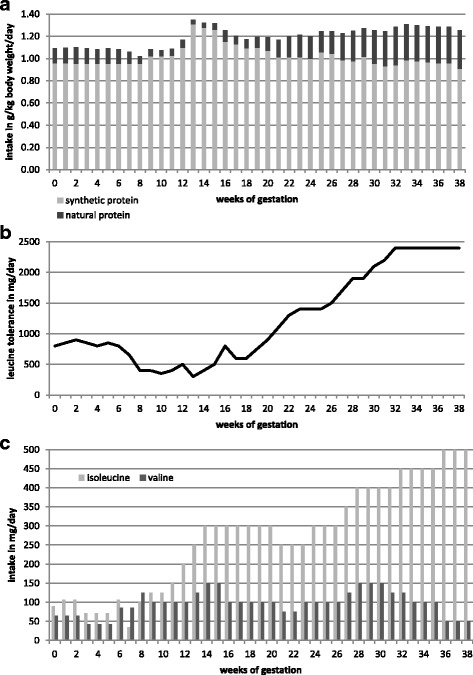
Details of dietary prescriptions during pregnancy. **a** Intake of total protein, natural protein and synthetic protein. **b** Leucine tolerance. **c** Supplementation of isoleucine and valine

**Fig. 2:**
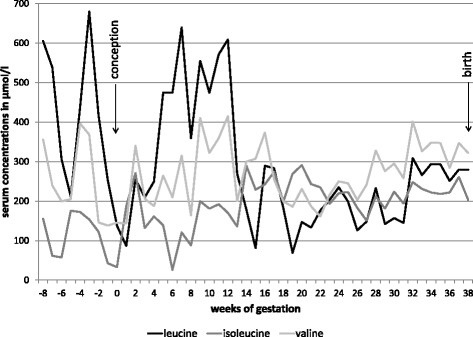
Concentrations of branched-chain amino acids in serum during pregnancy

The patient presented in the metabolic outpatient clinic as early as week 7 of gestation. This was the first pregnancy of the patient. No relevant problems such as nausea or hyperemesis gravidarum occurred during the first trimester. Pre-pregnancy weight was 74 kg, and body mass index was 27.2 kg/m^2^. Weight gain during pregnancy was 15 kg (0 kg during 1st trimester, 7 kg during 2nd trimester, 8 kg during 3rd trimester). Serum amino acids were monitored weekly throughout pregnancy and the diet was adapted regularly according to BCAA concentrations. Target concentrations of BCAAs were: leucine 100-300 μmol/L, isoleucine 100-300 μmol/L and valine 200-400 μmol/L. During the first trimester, these goals were hard to achieve and leucine levels ranged between 87 and 609 μmol/L. From the second trimester protein tolerance increased significantly to up to 2400 mg (= 27 mg/kg/d) leucine per day prior to delivery. Details of the prescribed diet including leucine intake, total protein, synthetic protein as well as isoleucine and valine supplementation are shown in Fig. 1. Additionally, examples of dietary protocols with different amounts of leucine are given in Additional file 1: Table S1, Additional file 2: Table S2, Additional file 3: Table S3. From the third month of pregnancy, a bedtime snack was introduced. The BCAA-free amino acid mixture was divided to 4 to 5 daily doses. BCAA levels during pregnancy are outlined in Fig. 2. Laboratory values including whole blood count, iron status, total protein, albumin, transaminases and creatinine were checked regularly and remained normal throughout pregnancy. A pregnancy-specific vitamin supplement containing folic acid, jodine, vitamin B1, B2, B6, B12, C, D3, E, biotin and niacin was given during the first trimester according to routine gynecological recommendations. From gestational week 15 the patient additionally received progesterone. Later in pregnancy, magnesium and iron were substituted to prevent premature contractions and pregnancy-related anemia, respectively, and omeprazole was added due to gastroesophageal reflux.

Pregnancy was uneventful, and no major problems occurred. Mild proteinuria was present within the second trimester, but resolved spontaneously. Fetal arrhythmia was noted in week 17, but considered non-pathological by a cardiologic specialist. During pregnancy the patient was followed by the metabolic team of the University Children’s Hospital for treatment of MSUD in collaboration with the Obstetric Department of the University Hospital. As the patient lived quite far from the metabolic center delivery was planned as Cesaerean section to ensure maximal safety for the patient. A treatment plan was tailored for the peripartal period, including infusion therapy to prevent perioperative catabolism, dietary management and necessary monitoring of mother and child. The Cesaerean section was performed in week 38. The patient was admitted 1 day in advance, and a triple-lumen central venous catheter was inserted. Few hours before surgery, when she had to be nil per os, parenteral nutrition containing 5 g glucose per kg body weight per day (G40%) and 1 g fat per kg body weight per day (20% intravenous fat emulsion) was given together with 1.5 l per day of an isotonic electrolyte infusion. Surgery was without complications, and the patient delivered a healthy female infant with a birth weight of 2795 g, birth length of 47 cm and a head circumference of 34 cm. Apgar scores at 5 and 10 min were 9 and 10, respectively. Newborn screening results were normal including that for MSUD.

On the day of delivery nutritional leucine intake was reduced to 200 mg/day due to concerns about postpartum catabolism and protein turnover associated with uterus involution. The dietary protocol with details on food intake on that day is shown in Additional file 3: Table S3. Oral intake, especially supplementation of the BCAA-free amino acid mixture was possible few hours after delivery. As the patient was in a good condition and able to take the targeted caloric amount orally, parenteral nutrition was already reduced to 50 and 25% within the following 3 days, and could be stopped on day 5 after birth. BCAA concentrations were measured daily during the postpartum period, and BCAA intake (protein intake, isoleucine and valine supplementation) was adjusted accordingly. Vigilance was checked every 8 h, and blood gases, electrolytes and glucose levels were monitored 2-3 times per day as were ketone bodies in urine. The patient was encouraged to take additional nonprotein calories orally from food and beverages. Lactation was well-established, and dietary leucine intake was gradually increased. The patient could be discharged from hospital on day 7 postpartum. The clinical course during breastfeeding remained stable with leucine levels ranging between 91 and 340 μmol/L. The leucine tolerance during lactation was 1700 mg/day (at 2 months after birth), around twice the pre-pregnancy intake. Her weight postpartum decreased by 9 kg within 2 months. Infant growth and development was normal at 7 months (body length 66 cm (22nd percentile), body weight 7460 g (40th percentile), head circumference 43 cm (47th percentile) and BMI 17,1 kg/m^2^(62nd percentile)).

## Discussion and conclusions

Adult patients with inborn errors of metabolism are a relatively new phenomenon. Medical progress with respect to newborn screening, early diagnosis and medical treatment including dietary management has resulted in an increasing number of patients with inborn errors of metabolism who nowadays reach child-bearing age. However, experience in the management of pregnancy, delivery and lactation is still limited for most disorders. Only nine cases of pregnancy in classical MSUD patients have been reported so far [4–10]. Details on these cases are displayed in Table 1. All were managed successfully and resulted in healthy offspring. Although there may be a publication bias in favor of cases with favorable outcome, the experience available so far suggests that pregnancy with successful outcome can be achieved.

**Table 1:** Overview on all pregnancies in MSUD mothers published in the literature

	MSUD subtype	presentation in metabolic outpatient clinic (week/month of gestation)	compliance during pregnancy	leucine tolerance before pregnancy (mg/kg/d)	natural protein tolerance before pregnancy	maximum leucine tolerance during pregnancy (mg/kg/d)	maximum protein tolerance during pregnancy	mode of delivery	peripartal infusion therapy	discharge from hospital (day postpartum)	offspring	breastfeeding	additional information	Reference
1	classical	2 months	good	n.a.	0.6-0.8 g/kg/d	n.a.	1.5 g/kg/d	spontaneous, 40 weeks of gestation	yes	n.a.	healthy	no	slowed fetal growth in week 37 with concomitant low BCAA levels, 9 days postpartum dizziness and lethargy, leucine level 1015 μmol/l	Van Calcar 1992 [5]
2	classical	6th week	good	n.a., 350-750	n.a.	2100	n.a.	spontaneous, 36 weeks of gestation	no	n.a.	healthy	yes	leucine peak of 1100 μmol/l on day 9 postpartum	Grünewald 1998 [8]
3	n.a.	24th week	good	n.a.	n.a.	n.a.	1.2 g/kg/d	spontaneous, 36 weeks of gestation	n.a.	day 16	healthy	n.a.	elevated BCAA levels postpartum, death of the mother on day 51 postpartum, bilateral pulmonary contusions and brain edema	Yoshida 2003 [7]
4	classical	10 weeks	poor	n.a.	15–35 g/d	n.a.	n.a.	spontaneous, 40 weeks of gestation	n.a.	day 8	healthy	n.a.	metabolic decompensation with several seizures and unconciusness in week 14 of gestation; mastitis on day 12 post partum, leucine level 549 μmol/l.	Tchan 2013 [10]
5	classical	27 weeks	poor	n.a.	30-50 g/d	n.a.	n.a.	emergency Caesarean section due to rupture of membranes and failure to progress, 41 weeks of gestation	n.a.	selfdischarged against medical advice on day 12	healthy	n.a.	Poor compliance with leucine levels typically 700–1300 μmol/l; acute febrile illness and threatened premature labour in week 28 of gestation; mineral, vitamin and protein deficiency diagnosed durig pregnancy	Tchan 2013 [10]
6	classical	10 weeks	good	600-900	n.a.	2400	n.a.	induced labour in week 37, chorioamnionitis and failure to progressafter 48 h, secondary Caesarean section	yes	day 10	healthy	yes	mild nausea and vomiting in early pregnancy; mild increase in blood pressure and concerns about preeclampsia in week 36; 4 episodes of elevated leucine levels up to 1082 μmol/L during lactation	Wessel 2015 [4]
7	classical	7th week	good	300-500	5 g/d	3000	30 g/d	spontaneous, 41 weeks of gestation	yes	day 5	healthy	yes	no complications	Heiber 2015 [9]
8	moderate	n.a.	n.a.	n.a.	30 g/d	n.a.	n.a.	n.a.	n.a.	n.a.	healthy	n.a.	same mother, first pregnancy of the mother not reported in deteil, but „was managed in a similar way to the second one“; persistent nausea throughout the pregnancy	Brown 2017 [6]
9	moderate	n.a.	good	n.a.	30 g/d	n.a.	60 g/d	spontaneous, 40 weeks of gestation	no	day 4	healthy	no		Brown 2017 [6]
10	classical	7th week	good	750	5-10 g/d	2400	30 g/d	Caesarean section, 38 weeks of gestation	yes	day 7	healthy	yes	no complications	this case

*n.a.* not available

It is known that pregnancy may be a challenge in the management of MSUD requiring careful monitoring and regular dietary adjustments. The pregnancy in our patient became known as early as week 7 of gestation. Amino acid concentrations were monitored at least weekly throughout pregnancy and lactation. Despite excellent compliance metabolic control was not easy to achieve during the first trimester and leucine levels were often above the target range, possibly due to an inadequate caloric intake. Grossly elevated leucine levels throughout the whole course of pregnancy have also been reported in 2 cases by Tchan et al. due to compliance problems [10]. Evidence on potential harmful effects of high concentrations of leucine, 2-oxoiocaproic acid and other MSUD related metabolites is very limited. Data from animal models are not available. However, all reported pregnancies with MSUD resulted in healthy newborns, making a teratogenic effect of MSUD metabolites unlikely. Nevertheless, subtle long-term effects cannot be excluded as none of the children was followed for more than 3 years, and standardized developmental testing was only performed in one infant at 7 months [4]. As it has been shown for healthy pregnancies as well as for MSUD pregnancies that amino acid concentrations in cord blood are 1.5 fold the maternal levels owing to active transport via the placenta it is recommendable to keep BCAA levels in pregnant MSUD patients as close to the normal range as possible [5, 8, 11].

As known from other cases leucine tolerance increased progressively from the second trimester. This can possibly be explained by both enhanced protein synthesis due to fetal growth and new capacity for BCAA metabolism gained from the liver of the heterozygous fetus [8]. In our case, leucine intake could be increased to 2400 mg/day prior to delivery, which was about 3 times the pre-pregnancy leucine tolerance. To minimize metabolic stress during delivery and the postpartum period leucine intake was reduced to 200 mg/day on the day of delivery and subsequently titrated up to 1700 mg/day during lactation based on BCAA concentrations in serum. The nutritional therapy was based on the recommendations published by Frazier et al. [12].

To provide maximal safety for mother and child we recommended that our patient should give birth in a specialized metabolic center. Primary Cesaerean section was proposed due to geographical distance between the patient’s place of residence and our university hospital. Alternatively, planned induction of labour can also be considered in case of good compliance of the patient and geographical proximity to the obstetric/ metabolic centre. Previous reports have shown that mothers with MSUD are at risk of developing high blood leucine concentrations especially in the postpartum period, possibly due to uterine involution. Three patients reported in the literature had leucine concentrations of more than 1000 μmol/L on day 9-10 postpartum [5, 8, 10], demonstrating that BCAA levels should be closely monitored during this period. Only one patient showed neurologic symptoms including dizziness and lethargy. In all cases leucine levels dropped spontaneously, and infusion therapy was not necessary. There is one report of maternal death at day 51 postpartum, however, it is unclear whether the patient died of metabolic decompensation or domestic violence [7].

Our case demonstrates that successful management of pregnancy in classical MSUD is possible. Metabolic decompensations can be prevented by an individually tailored treatment plan including protein restriction, adequate caloric supply and prevention of catabolism. Optimal management for pregnant patients with inborn errors of metabolism requires close collaboration of all specialists involved and delivery should be arranged in a center with experience in the treatment of inborn errors of metabolism.

## Additional files

Additional file 1: **Table S1.** MSUD diet prior to conception (leucine intake 600 mg/day). (DOCX 15 kb)

Additional file 2: **Table S2.** MSUD diet at the end of pregnancy (leucine intake 2400 mg/day). (DOCX 15 kb)

Additional file 3: **Table S3.** MSUD diet at the day of delivery (leucine intake 200 mg/day, additional to a high-caloric infusion therapy). (DOCX 15 kb)

## Abbreviations

BCAA: Branched-chain amino acids
MUSD: Maple syrup urine disease
