# Supplementary material for: Successful pregnancy in maple syrup urine disease: a case report and review of the literature
Source: Nutr J. 2018 May 12;17:51. doi: 10.1186/s12937-018-0357-7 (PMC5948788; doi:10.1186/s12937-018-0357-7)
Supplement: Supplementary file 1 — Table S1. MSUD diet prior to conception (leucine intake 600 mg/day). (DOCX 15 kb) [file 12937_2018_357_MOESM1_ESM.docx]

**Table S1: MSUD diet prior to conception (leucine intake 600mg/day)**

| **Meal** | **Calories (kcal)** | **Protein (g)** | **Leucine (mg)** |
| --- | --- | --- | --- |
| **Breakfast** |  |  |  |
| 60g low protein flakes | 190 | 0,2 | 9 |
| 120ml low protein milk | 79 | 0,0 | 0 |
| 100g apple | 57 | 0.3 | 16 |
| 130ml MSUD liquid amino acid mixture | 92 | 15 | 0.0 |
| **Lunch** |  |  |  |
| 180g potatoes | 131 | 3.4 | 223 |
| 25ml cream (30%) | 74 | 0,6 | 55 |
| 150g carrots | 59 | 1.3 | 65 |
| 10g butter | 74 | 0.1 | 7 |
| 70g salad | 11 | 0.7 | 57 |
| 5ml vegetable oil for dressing | 41 | 0 | 0 |
| 29g MSUD amino acid mixture | 92 | 20.3 | 0.0 |
| **Snack** |  |  |  |
| 100g pear | 58 | 0.5 | 36 |
| 100g strawberry | 78 | 0.7 | 44 |
| 10g sugar | 40 | 0.0 | 0.0 |
| 30g low protein biscuits | 140 | 0.1 | 6 |
| 150ml apple juice | 77 | 0.3 | 28 |
| 130ml MSUD liquid amino acid mixture | 92 | 15 | 0,0 |
| **Dinner** |  |  |  |
| 50g low protein bread | 134 | 0.3 | 10 |
| 10g butter | 74 | 0.1 | 7 |
| Two slides (à 20 g) protein-free cheese | 111 | 0.0 | 0.0 |
| 80 g tomato | 15 | 0.8 | 24 |
| 80 g cucumber | 10 | 0.5 | 20 |
| 29g MSUD amino acid mixture | 92 | 20.3 | 0.0 |
| Total: | 1820 kcal/d | 80.3g/d | 607 mg Leu/d |
| Total Protein 80.3g/d (19%), Fat 51g (26%); Carbohydrates 231g (54%); Fibres 16.8g/d (2%) | | | |
