# Supplementary material for: Successful pregnancy in maple syrup urine disease: a case report and review of the literature
Source: Nutr J. 2018 May 12;17:51. doi: 10.1186/s12937-018-0357-7 (PMC5948788; doi:10.1186/s12937-018-0357-7)
Supplement: Supplementary file 2 — Table S2. MSUD diet at the end of pregnancy (leucine intake 2400 mg/day). (DOCX 15 kb) [file 12937_2018_357_MOESM2_ESM.docx]

**Table S2: MSUD diet at the end of pregnancy (leucine intake 2400mg/day)**

| **Meal** | **Calories (kcal)** | **Protein (g)** | **Leucine (mg)** |
| --- | --- | --- | --- |
| **Breakfast** |  |  |  |
| 60g normal bread | 175 | 6.1 | 419 |
| 10g margarine | 71 | 0.0 | 2 |
| 35 g jam | 91 | 0.1 | 6 |
| 25g honey | 77 | 0.1 | 7 |
| 30ml protein free milk | 20 | 0.0 | 0 |
| 29g MSUD amino acid mixture | 92 | 20.3 | 0.0 |
| **Lunch** |  |  |  |
| 220g potatoes | 161 | 4.2 | 273 |
| 15ml vegetable oil | 124 | 0.0 | 0.0 |
| 80g green beans | 30 | 2.0 | 131 |
| 70g salad | 11 | 0.7 | 57 |
| 5ml vegetable oil for dressing | 41 | 0 | 0 |
| 29g MSUD amino acid mixture | 92 | 20.3 | 0.0 |
| **snack** |  |  |  |
| 120g apple | 68 | 0.4 | 19 |
| 120g melon | 47 | 0.7 | 30 |
| 50g banana | 47 | 0.6 | 43 |
| 50g quark (20%) | 55 | 6.1 | 645 |
| 20g low protein biscuits | 93 | 0.1 | 4 |
| 15g sugar | 61 | 0.0 | 0.0 |
| 150ml apple juice | 77 | 0.3 | 28 |
| 29g MSUD amino acid mixture | 92 | 20.3 | 0.0 |
| **Dinner & bedtime snack** |  |  |  |
| 60g normal bread | 175 | 6.1 | 419 |
| 20g margarine | 142 | 0.0 | 4 |
| 15 g cream cheese (28%) | 41 | 0.4 | 30 |
| 15 g salami | 70 | 3.9 | 27 |
| 50 g carrots | 20 | 0.4 | 22 |
| 100g zucchini | 24 | 2.1 | 152 |
| 80g celery | 20 | 1.2 | 75 |
| 5ml vegetable oil | 44 | 0.0 | 0.0 |
| 50 g cranberries (compote) | 17 | 0.3 | 30 |
| 29g MSUD amino acid mixture | 92 | 20.3 | 0.0 |
| Total: | 2165kcal/d | 117.0g/d | 2421 mg Leu/d |
| Total protein 117g/d (22%), Fat 70g/d (29%); Carbohydrates 251g/d (47%) Fibres 19.8g/d (2%) | | | |
