# Supplementary material for: Successful pregnancy in maple syrup urine disease: a case report and review of the literature
Source: Nutr J. 2018 May 12;17:51. doi: 10.1186/s12937-018-0357-7 (PMC5948788; doi:10.1186/s12937-018-0357-7)
Supplement: Supplementary file 3 — Table S3. MSUD diet at the day of delivery (leucine intake 200 mg/day, additional to a high-caloric infusion therapy). (DOCX 15 kb) [file 12937_2018_357_MOESM3_ESM.docx]

**Table S3: MSUD diet at the day of delivery (leucine intake 200mg/day, additional to a high-caloric infusion therapy)**

| **Meal** | **Calories (kcal)** | **Protein (g)** | **Leucine (mg)** |
| --- | --- | --- | --- |
| **Breakfast** |  |  |  |
| 50g low protein bread | 134 | 0.3 | 10 |
| 10g butter | 74 | 0.1 | 7 |
| 30 g jam | 78 | 0.1 | 5 |
| 29g MSUD amino acid mixture | 92 | 20.3 | 0.0 |
| **Lunch** |  |  |  |
| 100g low protein noodles | 351 | 0.5 | 44 |
| 60g sweet pepper | 14 | 0.6 | 38 |
| 70ml tomato sauce | 58 | 1.4 | 18 |
| 25g protein-free cheese | 69 | 0.0 | 0.0 |
| 5ml vegetable oil for dressing | 41 | 0 | 0 |
| 29g MSUD amino acid mixture | 92 | 20.3 | 0.0 |
| **Snack** |  |  |  |
| 200ml coffee | 4 | 0.4 | 34 |
| 30ml protein-free milk | 20 | 0.0 | 0.0 |
| 100g apple | 65 | 0.3 | 16 |
| 29g MSUD amino acid mixture | 92 | 20.3 | 0.0 |
| **Dinner & bedtime snack** |  |  |  |
| 40g low protein bread | 107 | 0.2 | 8 |
| 5g butter | 37 | 0.0 | 3 |
| 30g protein-free cheese | 83 | 0.0 | 0.0 |
| 5ml vegetable oil | 44 | 0.0 | 0.0 |
| 120g cucumber | 16 | 0.7 | 30 |
| 29g MSUD amino acid mixture | 92 | 20.3 | 0.0 |
| Total: | 1560kcal/d | 85.9g/d | 213 mg Leu/d |
| Total Protein 85.9g/d (22%); Fat 38g/d (22%); Carbohydrates 205g/d (54%), Fibres 13.7g/d (2%) | | | |
